# Supplementary material for: Student Evaluation of Distance Learning during the COVID-19 Pandemic: A Cross-Sectional Survey on Medical, Dental, and Healthcare Students at Sapienza University of Rome
Source: Int J Environ Res Public Health. 2022 Aug 19;19(16):10351. doi: 10.3390/ijerph191610351 (PMC9407842; doi:10.3390/ijerph191610351)
Supplement: Supplementary file 1 [file ijerph-19-10351-s001.zip › ijerph-1861249-supplementary.pdf]

SURVEY ANSWERS

Q1 - How do you evaluate your degree of preparation, at the beginning of the pandemic, in the use of informatic platforms (Google Meet, Classroom, Zoom, etc.) for DL?

a) Superb: 17.74% (66)
b) Good: 45.70% (170)
c) Discrete: 23.93% (89)
d) Poor: 12.63% (47)

Q2 - Were the electronic devices in your possession (or that of your family) at the beginning of the pandemic adequate (in number and/or characteristics) for the DL?

a) Yes: 77.15% (287)
b) No, I had to buy new devices: 20.70% (77)
c) No, but I could not afford the purchase of new devices: 2.15% (8)
d) No, but I was provided by the university on a free loan basis: 0% (0)

Q3 - With which device did you mainly follow the lessons in telematic mode?

a) PC: 86.56% (322)
b) Tablets: 9.14% (34)
c) Smartphone: 4.30% (16)

Q4 - How do you rate the quality of the internet connection of your home network?

a) Superb: 21.77% (81)
b) Good: 52.96% (197)
c) Discrete: 17.74% (66)
d) Poor: 7.53% (28)

Q5 - Have you had difficulty taking DL lessons due to connection problems?

a) Yes: 31.45% (117)
b) No: 68.55% (255)

Q6 - Have you had/do you have a quiet and adequate home environment for DL lessons?

a) Yes: 76.34% (284)
b) No: 23.66% (88)

Q7 - Has the presence of family members and/or roommates made it more difficult to follow the DL?

a) Yes: 46.77% (174)
b) No: 53.23% (198)

Q8 - From the beginning of the pandemic to today, how has your performance (average of grades) changed?

a) Increased: 17.74% (66)
b) Decreased: 17.74% (66)
c) Remained substantially unchanged: 64.52% (240)

Q9 - How has DL affected the progress of your studies?

a) It slowed it down: 25.27% (94)
b) It speeded it up: 26.61% (99)
c) It did not have a significant impact: 48.12% (179)

Q10 - Do you think that DL has had a negative effect on your professional preparation/maturation?

a) Definitely yes: 21.51% (80)
b) More yes than no: 26.88% (100)
c) More no than yes: 36.02% (134)
d) Definitely not: 15.59% (58)

Q11 - Do you think dad had a negative effect on your motivation to study?

a) Definitely yes: 33.33% (124)
b) More yes than no: 22.04% (82)
c) More no than yes: 24.73% (92)
d) Definitely not: 19.89% (74)

Q12 - Do you think that the suspension of internships (where provided) has produced significant gaps in your preparation?

a) Definitely yes: 50% (186)
b) More yes than no: 27.42% (102)
c) More no than yes: 8.60% (32)
d) Definitely not: 4.30% (16)
e) No internships: 9.68% (36)

Q13 - How do you judge the overall attitude of teachers to DL?

a) Very good: 13.71% (51)
b) Discrete: 47.58% (177)
c) Acceptable: 22.58% (84)
d) Poor: 16.13% (60)

Q14 - Do you think that an oral exam in telematic mode is adequate for a correct evaluation of your preparation?

a) Yes: 77.96% (290)
b) No: 22.04% (82)

Q15 - How has the DL affected active participation in lessons (interventions, questions, etc.)?

a) It reduced participation: 59.41% (221)
b) It encouraged participation: 13.17% (49)
c) It did not significantly affect participation: 27.42% (102)

Q16 - Do you think that the DL has facilitated the sharing of teaching material (handouts, slides, articles, etc.) by teachers)?

a) Yes: 78.49% (292)
b) No: 21.51% (80)

Q17 - How many hours a day did you spend on average at your PC/tablet/smartphone during DL periods?
a) Less than 3 hours: 5.37% (20)
b) Between 3 and 6 hours: 35.22% (131)
c) More than 6 hours: 59.41% (221)

Q18 - Have you experienced one or more of the following disorders due to the prolonged use of computer devices? (Multiple answers question)
a) Visual fatigue (e.g. burning, tearing, redness, visual tiredness): 64.78% (241)
b) Musculoskeletal disorders (e.g. cervicobrachialgia, low back pain, etc.): 39.52% (147)
c) Tiredness (e.g. headache, psychological and psychosomatic disorders): 83.06% (309)
d) Other: 16.04% (61)

Q19 - How do you judge the DL with respect to face-to-face teaching in terms of psycho-physical stress?
a) Most stressful: 29.84% (111)
b) Less stressful: 45.97% (171)
c) Indifferent: 24.19% (90)

Q20 - What is your overall assessment of university DL in the light of your personal experience?
a) Positive: 63.44% (236)
b) Negative: 36.56% (136)

Q21 - What do you think are the main advantages of DL? (Multiple answers question)
a) Avoid travel: 90.32% (336)
b) Reduces stress: 33.06% (123)
c) Economic savings: 56.99% (212)
d) Time savings: 76.08% (283)
e) Greater flexibility: 47.85% (178)
f) Greater interaction with teachers: 8.33% (31)
g) Other: 1.8% (7)

Q22 - What do you think are the main disadvantages of DL? (Multiple answers question)
a) Internet connection quality: 44.89% (167)
b) Domestic distractions: 57.26% (213)
c) Lack of space: 12.37% (46)
d) Lack of adequate informatic devices: 12.90% (48)
e) Poor interaction with other students: 80.38 % (299)
f) Poor interaction with teachers: 51.61% (192)
g) Difficulty in using the platforms for DL: 8.87% (33)
h) Lower performance in exams: 20.16% (75)
i) Other: 1.61% (6)

Q23 - Which of the following activities would you like to attend in the future in DL modality? (Multiple answers question)
a) Frontal lessons: 43.82% (163)

b) Group work: 19.89% (74)
c) Elective didactic activities: 32.26% (120)
d) Seminar: 52.15% (194)
e) None: 19.09% (71)
f) Other: 2.42% (9)
